# Immune Reactivation by Cell-Free Fetal DNA in Healthy Pregnancies Re-Purposed to Target Tumors: Novel Checkpoint Inhibition in Cancer Therapeutics

**DOI:** 10.3389/fimmu.2015.00424

**Published:** 2015-08-26

**Authors:** Elizabeth Ann L. Enninga, Wendy K. Nevala, Shernan G. Holtan, Svetomir N. Markovic

**Affiliations:** ^1^Mayo Graduate School, Mayo Clinic, Rochester, MN, USA; ^2^Department of Hematology, Mayo Clinic, Rochester, MN, USA; ^3^Department of Transplantation, University of Minnesota, Minneapolis, MN, USA; ^4^Department of Oncology, Mayo Clinic, Rochester, MN, USA

**Keywords:** cell-free fetal DNA, circulating tumor DNA, immunotherapy, inflammation, toll like receptors

## Abstract

The role of the immune system in cancer progression has become increasingly evident over the past decade. Chronic inflammation in the promotion of tumorigenesis is well established, and cancer-associated tolerance/immune evasion has long been appreciated. Recent developments of immunotherapies targeting cancer-associated inflammation and immune tolerance, such as cancer vaccines, cell therapies, neutralizing antibodies, and immune checkpoint inhibitors, have shown promising clinical results. However, despite significant therapeutic advances, most patients diagnosed with metastatic cancer still succumb to their malignancy. Treatments are often toxic, and the financial burden of novel therapies is significant. Thus, new methods for utilizing similar biological systems to compare complex biological processes can give us new hypotheses for combating cancer. One such approach is comparing trophoblastic growth and regulation to tumor invasion and immune escape. Novel concepts regarding immune activation in pregnancy, especially reactivation of the immune system at labor through toll like receptor engagement by fetal derived DNA, may be applicable to cancer immunotherapy. This review summarizes mechanisms of inflammation in cancer, current immunotherapies used in the clinic, and suggestions for looking beyond oncology for novel methods to reverse cancer-associated tolerance and immunologic exhaustion utilizing mechanisms encountered in normal human pregnancy.

## Introduction

Cancer is the complex orchestration of tumor and supporting stroma, immune mediators, and angiogenic factors that result in growth and metastasis of tumor cells, which lead to organ dysfunction and death if not successfully treated. What begins as a single cellular mutation in an oncogene and/or a tumor suppressor undergoing repeated insult culminates into a large, heterogeneous population of tumor cells that can migrate to distant sites, invade healthy tissues, and evade the host immune defenses. This last concept – the evasion of the host immune system – has become an increasingly recognized topic and therapeutic target in cancer biology. If the cellular machinery inside the cell cannot fix the mutations or cause apoptosis, the immune system must work to eradicate the developing cancer. However, tumor cells are “self,” making it more challenging for natural killer (NK) cells, cytotoxic T lymphocytes (CTLs), and other cellular effectors involved in immunosurveillance to target tumor cells compared to cells infected with a virus. Matzinger’s danger model states that the immune system is more concerned with damage (acute inflammation) signals than foreign antigens when eliciting a response (1). Since immune cells become tolerized by the tumor, they might not respond to danger signals the way a healthy immune system would. This interesting immunologic paradox also exists in the setting of pregnancy, where the mother is continually exposed to haploidentical “foreign” cells from the fetus but does not mount an immunologic attack that would be deleterious to the pregnancy. Tumor cells may mimic trophoblastic cells of the placenta in that they downregulate danger signals while increasing expression of immunosuppressive mediators (2, 3).

Determining new methods to elicit strong, specific, and durable anti-tumor immune responses is the focus of many laboratories. However, a number of barriers to successful immunotherapy exist. The first barrier is the impaired baseline immunologic function of cancer patients, even before they receive any therapy. For example, patients with advanced melanoma exist in a state of systemic chronic inflammation, which is driven, in part, by high levels of vascular endothelial growth factor (VEGF)-A secreted by the tumor to suppress the cancer targeting activity of immune cells (4, 5). The second barrier to successful immunotherapy is to determine which patients benefit from treatment. Analyzing blood from 21 patients on multiple vaccine trials who responded versus those that did not, researchers observed that melanoma patients who had a complete response were producing Th1-associated cytokines such as tumor necrosis factor (TNF) and interferon gamma (IFN-γ), unlike patients who had no response to the vaccine (6). It is well established that patients who have tumor-infiltrating lymphocytes (TILs) at the site of their malignancy tend to have better prognosis (7–10), and those who lack an organized immune response to melanoma have an extremely poor prognosis (11). The third barrier to successful immunotherapy is understanding when and how to couple immune-based therapies with standard cytotoxic chemotherapies or molecularly targeted treatments. Clinical and experimental data from our group and others have thus provided ample evidence that enriching our understanding of the host immune system’s interaction with malignancy is paramount to improving outcomes, regardless of mutational status and availability of targeted agents (12). The positive synergy accomplished by combining targeted therapy (i.e., BRAF or MEK mutations) with immunotherapy could also provide promising results and is currently being tested in a phase I clinical trial (NCT01767454) (13, 14). In this review, we will discuss the significance of immunity in many different cancers, including melanoma, and current methods to modulate it. Then, we transition to immunologic mechanisms in pregnancy exploited by tumors, and conclude with emerging data regarding the potential benefit of cell-free (fetal) nucleic acids in the reconstitution and prolongation of anti-tumor immunity.

## Current Knowledge and Methods in Cancer Biology

### Biology of chronic inflammation in cancer

Clinical and experimental data have revealed that patient outcomes in advanced cancers are strongly influenced by the type of immune response that is established prior to initial treatment. There are two important types of responses in cancer: acute (anti-tumor) and chronic (protumor) inflammation. Dr. William Coley was a pioneer in immunotherapy who utilized heat killed bacteria named “Coley’s toxins” to induce an acute inflammatory response in sarcoma patients. Coley’s toxins resulted in 5–10+ years of survival for ~50% of patients (15). This was the first clinical evidence that an acute inflammatory response will destroy tumor cells. After exposure to foreign antigen, innate cells such as macrophages and dendritic cells (DCs) travel to the site of infection and begin processing and presenting antigens to adaptive immune cells. Adaptive immune cells, specifically T and B lymphocytes, with specificity against the antigen then undergo expansion until that antigen is eliminated. In the setting of cancer, an acute inflammatory response causes destruction of the tumor through the activation of a Th1 T-helper cell response driven by IFN-γ and tumor killing by CTLs and NK cells. The expansion of M1, or classically activated macrophages, continues to activate other lymphocytes against the tumor through secretion of IFN-γ and presentation of tumor antigens (16, 17). Thus, immunosurveillance in an immune competent host can eliminate a majority of transformed cells before they induce malignancy (18).

If there is a prolonged (or suboptimal) exposure to the foreign antigen, a chronic inflammatory response is generated, which is supportive of tumor development and growth (19). In chronic inflammation, Th2 T-helper cells promote anergy, a loss of T-cell mediated cytotoxicity and B-cell activation through the secretion of interleukin (IL)-4, IL-5, IL-6, IL-10, and IL-13 (20). Regulatory T-cells (Tregs) expand and migrate to the site of the tumor and suppress DC, CTL, and NK cell anti-tumor effects (21). High numbers of FOXP3+ Tregs in the tumor were found to correlate with disease stage and poor overall survival (22). Tumor-associated macrophages (TAMs), or M2 alternatively activated macrophages, promote tumor progression through their ability to regulate VEGF and angiogenesis (23, 24). M2 polarization is triggered by chronically activated B-cell secretion of granulocyte-macrophage colony stimulating factor (GM-CSF), IL-6, and IL-10 (25). Myeloid derived suppressor cells (MDSCs) bind directly to CTLs and inhibit their anti-tumor effects through nitric oxide (NO) secretion (26). MDSCs also contribute to tumor blood vessel formation and metastasis through the production of matrix metalloproteinase (MMP)-9, which releases high levels of VEGF into the bloodstream (27, 28). These series of events work in concert to create a positive feedback loop that supports, rather than inhibits, tumor growth.

The hypothesis that tumors arise from sites of chronic inflammation was initiated by Virchow in 1863 and increasing amounts of evidence have supported his claim (29–32). Disease progression, metastatic spread to lymph nodes, tumor size, and patient survival correlate with high levels of CD4+ T-helper cells and low levels of CD8+ CTLs at the primary tumor in many types of cancers (33–37). Interestingly, transcription factor NF-κB was determined to be crucial for inflammation, and more recently, tumorigenesis as numerous cancer types show constitutive activation of NF-κB (38–41). Moreover, *Helicobacter pylori* infection is one of the main risk factors for gastric cancer and is believed to promote tumorigenesis through NF-κB activated transcription of IL-1, IL-6, IL-8, TNF-α, and cyclooxygenase-2 (COX2), which are all mediators of chronic inflammation (42, 43). Finally, chronic viral infections such as human papillomavirus (HPV) and hepatitis (both B and C) have been directly linked to cervical cancer, head and neck cancer, and liver cancer, respectively (44, 45). A case-control study conducted in the United States found that long-term use of non-steroidal anti-inflammatory drugs, as means to dampen chronic inflammation, decreased a person’s risk of developing melanoma by almost 50% (46). Altogether, mediators of chronic inflammation support the tumor’s ability to proliferate, invade, and migrate within the host promoting tumor cell survival.

### Therapeutics designed to enhance immunity against cancer

Many strategies exist to treat patients with various types of cancer. Targeting and destroying tumors using the host’s immune system is the basic principle of modern cancer immunotherapy. However, many patients do not respond to immunotherapy, the drugs are costly, and patients may suffer immunologic adverse events (AEs) that can be severe or life threatening. Table 1 summarizes results from clinical trials and the toxicities associated with therapy. Checkpoint inhibitors have revolutionized immunotherapy and are considered one of the most effective therapies for utilizing the immune system against tumors. Examples include anti-cytotoxic T lymphocyte antigen 4 (CTLA-4), anti-program death 1 (PD-1), and anti-program death ligand 1 (PD-L1) reviewed by Topalian et al. (47). The use of antibodies to block proteins known to promote tumor growth is of significant current interest in cancer therapy. Many of these drugs have shown to induce a response as a single agent or in combination with chemotherapy. Anti-VEGF)-A, anti-human epidermal growth factor receptor 2 (HER2/neu), and anti-CD20 are a few monoclonal antibodies used in oncology, but there are many more being studied (48). Immune-stimulating vaccines have also been developed for cancer patients. Therapeutic vaccines require a tumor specific antigen and an activation signal (immune adjuvant), such as a toll like receptor (TLR) agonist, in order to stimulate an immune response against an already established tumor. Common tumor antigens include melan-A, NY-ESO-1, B7C, and MAGE-1 (49–52). However, the challenge with many of these peptides is that they can be easily cleared without activating DCs. In addition, tumor antigen heterogeneity and changing expression of these antigens makes targeting ineffective. The most successful cancer vaccines include Provenge and Gardasil. Yet, the challenges with developing therapeutic vaccines include the many differences that are documented between trials, including vaccine strategy, antigen dose, tumor and patient heterogeneity, severity of disease, and vaccine adjuvants, which can all confound the results. These variables must be considered when developing therapeutic vaccines and testing their efficacy in clinical trials. Adoptive cell transfer (ACT) is another modality of cancer immunotherapy where cells, which can be unmanipulated, antigen-specific, or stimulated, are utilized to kill cancer cells in lymphodepleted patients. ACT has been successful at breaking tolerance in many cancers. Chimeric antigen receptor (CAR) therapy utilizes both targeting antibodies and cytotoxic CD8 T-cells for destroying cancer cells in a similar manner as ACT. For CAR therapy, T-cells are collected from cancer patients, expanded *in vitro* and their receptors are modified to more specifically target the tumor when given back to the patient (53). Despite some of these incredible response rates, ACT is expensive, requires that the patient have adequate lymphocytes for collection, needs specialized manufacturing facilities, regulatory hurdles, and is time prohibitive (54).

**Table 1:** **Therapeutic efficacy and related toxicities of drugs developed for cancer treatment**.

Therapeutic strategy	Target	Clinical benefit	Toxicity	Reference
**IMMUNOTHERAPY**				
Ipilimumab	Anti-CTLA-4	Increased OS from 6.4 to 10 months	15% had grade 3 or 4 AE	(55)
Pembrolizumab	Anti-PD-1	Response rate of 38%	Grade 1 or 2 AE	(56)
Ipilimumab + Nivolumab	Anti-CTLA-4 plus Anti-PD-1	Objective response 53%	50% had grade 3 or 4 AE	(57)
BMS-93655	Anti-PD-L1	Objective response 6%–17%	9% had grade 3 or 4 AE	(58)
**MONOCLONAL ANTIBODIES**				
Trastuzumab	Anti-HER2/neu	Increased OS from 20.3 to 25.1 months	27% had cardiac toxicity	(59)
Bevacizumab	Anti-VEGF	Increased OS from 15.6 to 20.3 months	Grade 3 hypertension	(60)
Rituximab	Anti-CD20	Clinical remission in 46% of patients	Grade 1 or 2 AE	(61)
**VACCINES**				
Provenge	PAP plus GM-CSF	Increased OS from 21.7 to 25.8 months	Grade 1 or 2 AE	(62)
Gardasil	HPV type 6, 11, 16, and 18	Efficacy was 98%	Grade 1 or 2 AE	(63)
Pemetrexed	MAGE-A3 + TLR4 + TLR9	No difference in OS	9% had grade 3 or 4 AE	(64)
Synthetic long-peptide	HPV-16 E6 plus HPV-16 E7	Response rate of 79%	Grade 1 or 2 AE	(65)
**ADOPTIVE CELL TRANSFER**				
T-cells	MART-1 or gp100	Response rate of 46%	Autoimmune events	(66)
Naïve T-cells	LY6K-177 peptides	Response rate of 22%	Grade 1 or 2 AE	(67)
Memory T-cells	MCF-7 cell lysate antigen	Increased OS to 33.8 months	No toxicity noted	(68)
CAR therapy	Modified CD19	Response rate of 90%	Cytokine release syndrome	(69)
CAR therapy	GD2 antigen	Median OS 931 days	15% had grade 1 or 3 AE	(70)

*AE, adverse event; HPV, human papillomavirus; OS, overall survival; PAP, prostatic acid phosphatase*.

## Novel Insights of Immune Tolerance from Pregnancy Biology

### Immunology of pregnancy: Breaking tolerance prior to labor

Exploring similar systems, such as immune tolerance of a haploidentical fetus, takes another approach to understanding complex immune regulation in tumorigenesis. The maternal immune system must effectively balance tolerance to paternal antigens while continuing to protect the mother from infection; failure to do so can result in negative pregnancy outcomes (71). Thus, a T-helper 2, or Th2, immune response has been defined as the dominant phenotype during pregnancy, and this phenotype is also evident in metastatic cancer (4, 72). The most profound similarity is that both must evade immune recognition and destruction by the host/maternal system while expressing foreign antigens (tumor versus paternal). Yet, how a fetal allograft is not rejected although the maternal immune system continues to be capable of responding to other foreign antigens remains of significant scientific interest today. Many have made the observation that tumors mimic the tolerant immune state required by a trophoblast for successful implantation (early pregnancy) (3, 73, 74). However, a modification occurs in late pregnancy leading to an acute inflammatory response driven by proinflammatory cytokines, hormones, and chemokines, which traffic effector leukocytes to the myometrium and initiate labor (75). The exact mechanism as to how labor is initiated in human beings remains unclear. Placental tissue derived from women with recurrent pregnancy loss showed signs of inflammation, such as elevated NK cells, thromboembolism, insufficient trophoblast invasion, and lesions compared to placental tissue from healthy pregnancies (76). In a murine model, complement activation decreased VEGF-A, which leads to miscarriage or growth restriction, and blocking complement activation reversed this affect (77). This demonstrates the necessity of careful immune regulation at the fetal–maternal interface in order to establish a viable pregnancy. Others have studied pregnant women and found that tumor-associated antigens (TAAs) like MUC1, HER2/neu, WT-1, and PRAME, which are highly expressed in placental tissue, elicit the strongest immune response during the first and second trimester, declining after delivery and the completion of nursing; however, history of delivery was not correlated with increasing immune responses (78). This suggests that although the fetus is being tolerated by the mother’s immune response, maternal immunity is still fully capable of reactivating when given a strong enough stimulus. Through better understanding of the mechanisms that drive immune reactivation (labor), we can gain valuable insight into possible tools to use for promoting tumor destruction in cancer patients.

### Fetal derived nucleic acids for immune activation

Cell-free fetal DNA (cff-DNA) and RNA strands are shed into the maternal system during pregnancy. The concentration of these nucleic acids increases in the circulation with the length of gestation (79, 80). During early gestation, the concentration of cff-DNA in plasma ranges between 0.022 and 0.46 ng/mL, which increases to 5.08ng/mL by late pregnancy (81). Clinically, these sequences have been used to determine chromosomal abnormalities or genetic mutations that a child might have inherited (82). A new hypothesis recently arose suggesting that the increase in cff-DNA at term could activate TLRs on maternal cells, which leads to the breakage of immune tolerance, the activation of innate immune cells, and finally the onset of labor (83). It has been well documented that foreign DNA is recognized by TLR9, and RNA is recognized by TLR3 on immune cells, which activate inflammatory processes (84, 85). TLR9 is found in the endosome of peripheral blood mononuclear cells, such as monocytes, macrophages, T, B, and NK cells, whereas TLR3 is expressed only in the endosomes of myeloid derived cells such as DCs and monocytes (86). Fetal RNA is derived from the placenta and was shown to be surprisingly stabile in plasma, although lower levels are detected compared to cff-DNA (87). Apoptosis of placenta and trophoblastic cells are believed to be the major source of cff-DNA in the maternal bloodstream (88, 89).

**Figure 1:**
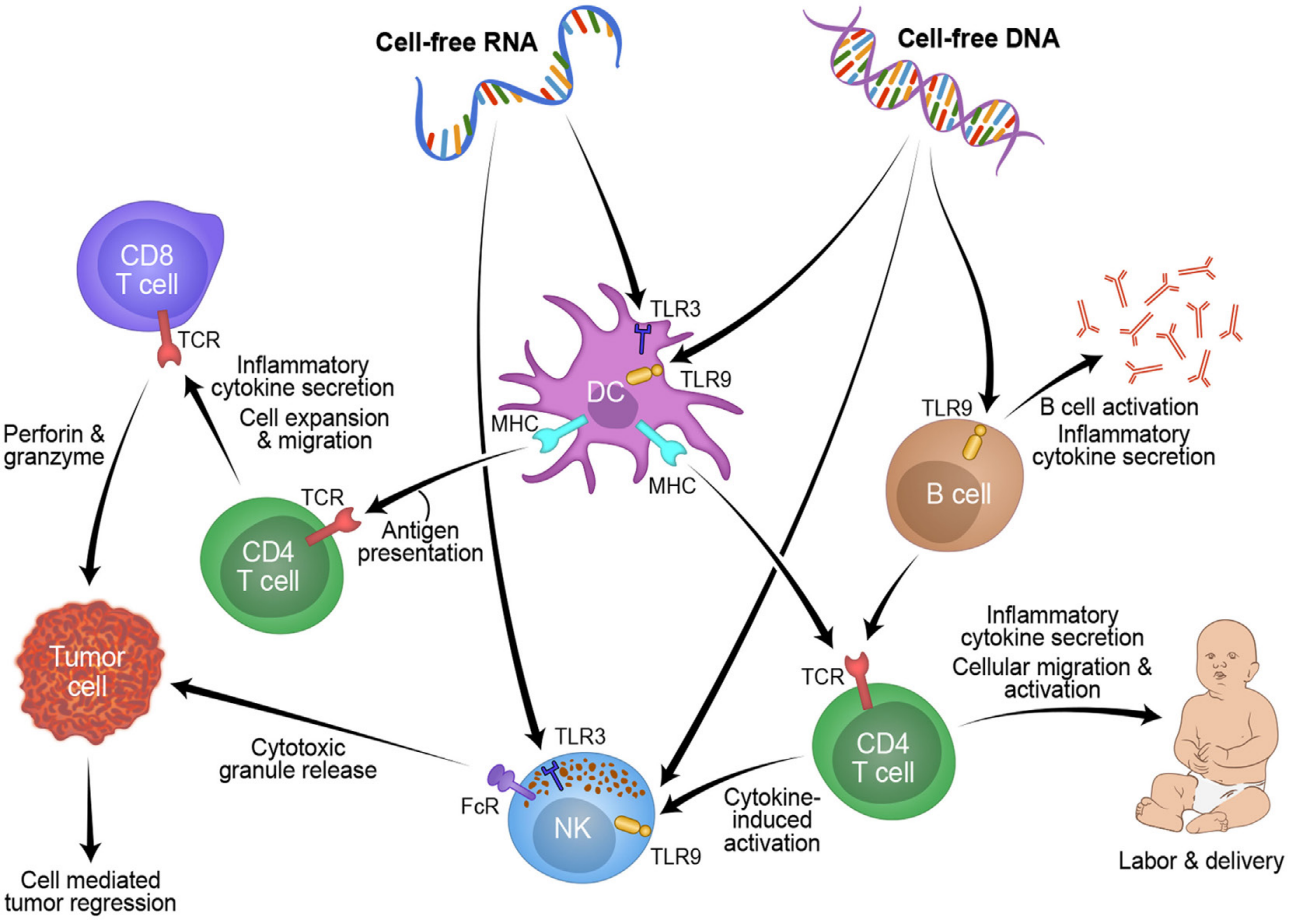
**Overview of how cell-free fetal derived RNA or DNA from pregnancy can activate an inflammatory immune response through toll like receptors, which could be applied to novel cancer treatments**.

Studying pregnancy disorders may also provide important mechanisms to reactivating inflammation for cancer patients. Three weeks before onset of symptoms, cff-DNA was increased between two- and five-fold in plasma of women suffering from preeclampsia compared to women with healthy pregnancies (90). Timing of the measurement and maternal health (BMI) are known confounders of cff-DNA measurement, thus another group found no difference between cff-DNA levels in preeclamptic women (91–93). From these discrepancies, a new idea was proposed: cff-DNA may be proinflammatory and high levels may send a danger signal to the maternal immune system (83). Using a mouse model of pre-term birth and preeclampsia, high levels of cff-DNA stimulated TLR9 to initiate acute inflammation, which caused fetal reabsorption at days 10–14, and knocking out TLR9 diminished this result (94). In general, TLRs and proinflammatory cytokines are overexpressed in women suffering from preeclampsia versus those with healthy pregnancies (95). In women suffering from hyperemesis gravidarum, cff-DNA levels were found to be 2.5-fold higher than healthy controls, and this is believed to be due to the increased activation of the maternal immune system, specifically the increased levels of cytotoxic T-cells and NK cells in the decidua and blood of the mother which target the haploidentical fetus (96, 97). Figure 1 shows a schematic of this possible process RNA and/or DNA could use to activate the labor process in pregnancy and how this mechanism could be manipulated for tumor rejection in cancer patients. Fetal DNA or RNA, at a critical concentration in maternal plasma, could be recognized by TLRs and processed by myeloid cells such as DCs or NK cells. These cells could then present this fetal antigen to lymphocytes resulting in an acute inflammatory response that leads to labor. This mechanism might also be applicable to reactivating anti-tumor immunity in cancer patients. Taken together, understanding of cff-DNA as possible proinflammatory mediator and predictive marker for disease onset during pregnancy could become a powerful resource for obstetrics research with translational value to oncology.

Sequence length, epigenetic modification, and/or confirmation of DNA may also be a critical factor when it comes to the ability of soluble DNA to activate immune cells. CpG motifs derived from *Escherichia coli* were the first sequences characterized, which bind and activate TLR9 in B-cells and plasmacytoid dendritic cells (pDCs) (84, 98). These unmethylated, often palindromic CpG motifs are more frequently found in bacterial DNA compared to DNA from human beings and short oligodeoxynuleotide (ODN) have been synthesized showing the same ability to activate TLR9 as bacterial DNA, suggesting sequence, not length, is imperative to achieve TLR9 activation (84). Interestingly, cff-DNA is comprised of mostly short sequences of 0.3 kb compared to maternal DNA (99). It is possible that this shorter length of cff-DNA may contain similar palindromic CpG sequences to the ODN sequences, which can effectively activate TLR9 signaling. Methylation typically occurs in CpG-rich regions (or promotor sites) in the mammalian genome and results in the suppression of transcription (100). Cff-DNA also has distinct methylation patterns based on differentiation during embryogenesis that are unlike the maternal genome (101). Poon et al. utilized the IGF2-H19 locus to distinguish chimeric fetal DNA from maternal since this region is only methylated on the paternal allele (102). Another example is the maspin gene promotor, which was found to be hypomethylated in placental tissue compared to hypermethylated in maternal blood cells (103). Since cff-DNA is likely derived from the placenta, utilizing methylation status in plasma could be a more effective way to distinguish fetal derived DNA irrespective of the sex of the fetus or polymorphisms. This finding is similar to unmethylated ODN sequences used to activate TLR9 signaling. When the ODN sequence was methylated, researchers found no immune stimulatory effects compared to the unmethylated sequence, demonstrating the importance of this epigenetic marker (84). Further research into whether these epigenetic markers make a difference in affinity for TLR binding and activation of innate immunity needs to be completed. Additionally, the folding pattern of cff-DNA may also be important for effective receptor triggering and signaling. DNA can self-assemble into many different structures based on nucleic acid abundance and/or intrinsic atom properties (104). Thus, specific folding patterns of nucleic acids could be better at activating innate immune receptors compared to other patterns. Taken together, understanding the multiple factors that could be pivotal to circulating DNA may help us better understand haploidentical cell-free DNA or RNA and how it could be utilized in cancer patients to activate anti-tumor effects.

### Circulating nucleic acids and toll like receptor signaling in cancer

Interestingly, there are cell-free nucleic acids found in healthy individuals and these levels were found to be elevated in blood of patients with many different cancers (105–109). It is unclear how this DNA gets into the bloodstream, but it is believed to be the by-product of macrophage engulfment of necrotic and/or apoptotic cells (110). Since tumors have areas of high necrosis, this hypothesis would explain the increase in circulating tumor (ct)-DNA fragments found in cancer patients. In addition, particle associated RNA was also found at increased levels in cancer patients compared to healthy individuals, although much less studied (111). The size of these DNA fragments is also important to note, varying from small fragments (70–200 bp) to large fragments around 21 kb (112). Serum from cancer patients has an average of 180 ng/mL of cell-free DNA compared to healthy subjects having an average of only 30 ng/mL (107, 113). Determining which DNA sequences have tumor origin versus background circulating DNA fragments is difficult and the use of these fragments for diagnostic or prognostic value remains controversial. In breast cancer, high levels of ct-DNA correlated with tumor size, grade, staging, lymph node status, and metastasis (105, 109). Survival was also reported to correlate with ct-DNA levels: breast cancer patients with high levels of ct-DNA in their blood had a lower OS than those with low levels of ct-DNA (114). Yet, others find no correlation between level of ct-DNA and survival in lung cancer or colorectal cancer (115, 116). Some of these differences could be due to the challenges and methods of isolating these short fragments including blood collection and processing methods, time elapsed between draw and isolation, and the isolation technique (117). Epigenetics of ct-DNA was also found to be important in the development of carcinogenesis, and new methods are being developed for measuring differentially methylated tumor DNA. Methylation studies have demonstrated transcriptional repression at CpG islands of tumor suppressor genes that lead to cancer progression (118). Thus, many new technologies are being developed to find hypermethylated promotors, especially proto-oncogenes regions, which are not present in healthy persons. CDKN2A, PARP-1, and GSTP1 are just a few genes that were found to be hypermethylated in ct-DNA and tumor tissue, and are being studied for biomarker use (119–121). In many instances, hypermethylation has been found to correlate with worse survival; therefore, many groups are working on development of epigenetic therapy, which has been reviewed by Jones and Baylin (122). Although the results of ct-DNA studies remain inconclusive, the promise of using a minimally invasive method to diagnosis and treat cancer patients makes it worth continually pursuing.

As suggested above, TLR signaling could be significant for the reactivation of immunity in pregnancy and possibly cancer. However, biology tells us that a patient’s cell-free DNA does not activate TLR signaling on their immune cells; if it did, autoimmunity would occur. Table 2 compares and contrasts cff-DNA to ct-DNA. Major known differences include methylation status and size; however, sequence and structure have yet to be considered as possible differences between the two. Overall, a better understanding of TLR adjuvants is critical to improve patient care. Clinically, there are several TLR agonists which have been tested for the treatment of cancer. TLR9 is expressed on chronic lymphocytic leukemia cells and will undergo apoptosis when given CpG ODN 2006 (TLR9 agonist) *in vitro* (123). TLR3 agonist bacillus Calmette–Guerin (BCG) given to mice prior to tumor injection, were less likely to develop tumors than their untreated littermates and this was due to the increase in TNF (124). Clinically, TLR agonists have not been as impressive as their pre-clinical results. In phase II studies of CpG ODN 2006, anti-tumor effects were modest in T-cell lymphoma and melanoma as a single agent (125, 126). BCG was approved for early stage bladder cancer after randomized studies showed that 88% of patients had a complete response and a reduction in tumor recurrence (127, 128). The challenge with development of TLR agonists includes understanding which TLRs are involved in protumor versus anti-tumor effects and how to target these agonists to the site of the tumor more effectively. Although the effects have been moderate, there is hope that combination with other drugs along with specifically targeting the anti-tumor T-cells will improve clinical efficacy (129). Immune responses are tightly regulated; just as TLR stimulation will activate responses it will also suppress them as to prevent autoimmunity. TLR7/8 and TLR4 agonists promote expression of negative co-stimulatory PD-L1 on DCs, which inhibits anti-tumor effects (130). However, a combined PD-1/PD-L1 blockade with a TLR3 agonist resulted in an increase of CD8 T-cell effectors and anti-tumor responses in a murine melanoma model (131). Maternal microenvironment may also play a key role in regulating responses to nucleic acids. During pregnancy, IL-27, a Th1 promoting cytokine, increases with length of gestation and decreases just after delivery in a very similar fashion to fDNA in maternal plasma (132). Thus, along with a critical concentration of DNA, other factors, such as known proinflammatory cytokines, are likely required to further drive activation of lymphoid and myeloid cells against a haploidentical fetus and an altered-self tumor.

**Table 2:** **Differences and similarities between fetal-derived and ­tumor-derived circulating DNA**.

Characteristic	Cell-free fetal DNA	Circulating tumor DNA
Methylation status	Hypomethylated	Hypermethylated
Size	~300 bp	70–200 bp or 21 kb+
Plasma concentration	Early: 0.02–0.46 ng/mL; Late: 0.46–5.08 ng/mL	180 ng/mL
Origin	Apoptosis of placenta or fetal cells	Necrosis of tumor cells

### Basic *in vitro* observations of cff-DNA and TLR activation

**Figure 2:**
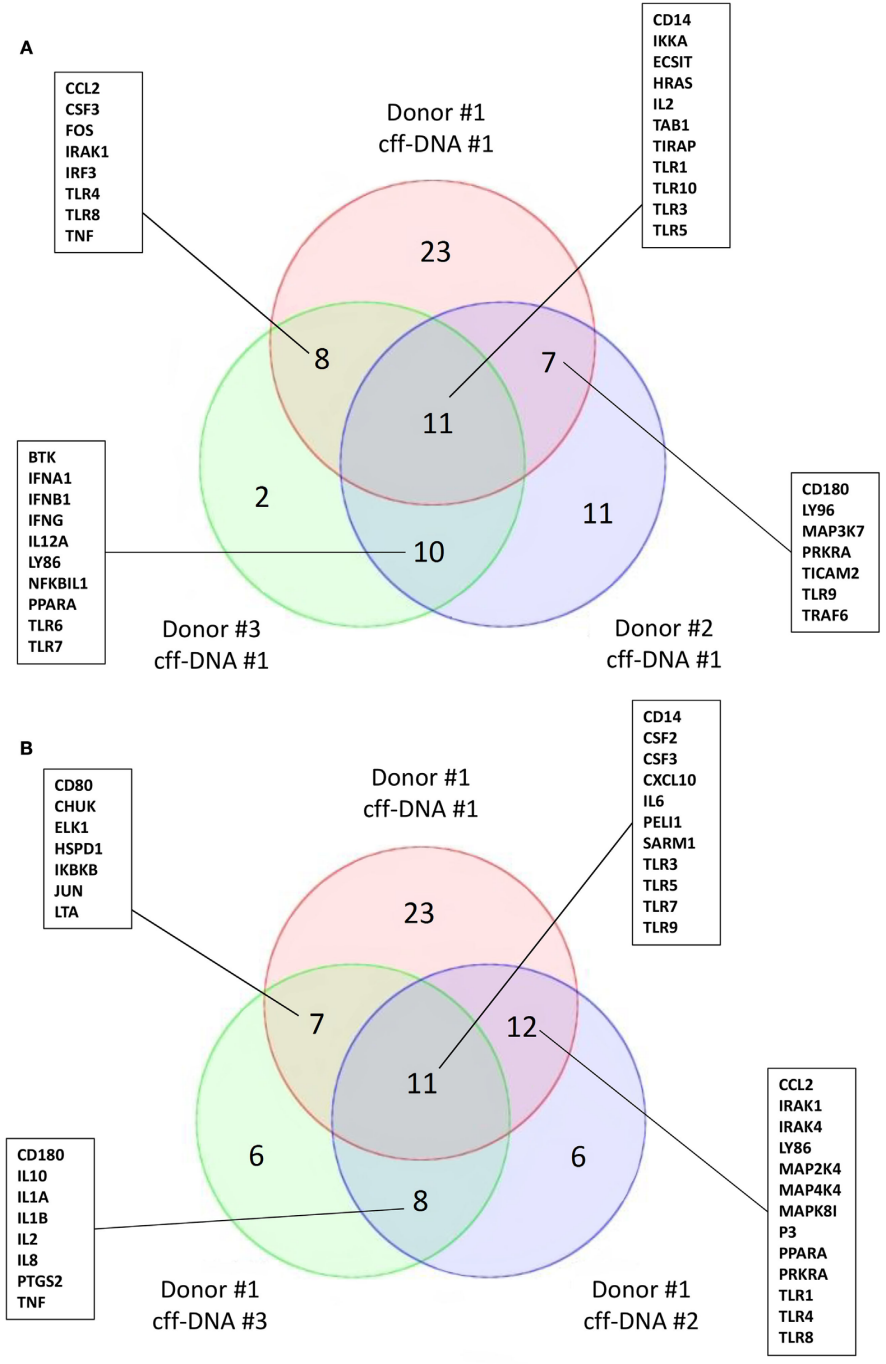
**Activation of TLR signaling cascade on CD14+ monocytes with addition of cff-DNA**. Venn diagrams showing similar genes involved in the TLR signaling pathway having a fold change cut off of 2. **(A)** Three different healthy CD14+ monocyte populations treated with the same cff-DNA. **(B)** One healthy CD14+ monocyte population treated with three different cff-DNAs.

To begin understanding if DNA might reactivate immunity at parturition, we isolated fDNA from the plasma of four pregnant women between 36 and 40 weeks of gestation using the Akonni Circulating DNA TruTip method (133). CD14+ monocytes isolated from healthy donors (males 36–44 years) were treated with 0.5 μg/mL of cff-DNA for 6 h before they were lysed and RNA was isolated per Qiagen’s RNeasy protocol. We added a much higher concentration of cff-DNA to the monocytes compared to what is biologically seen, to address the fact that our DNA population is not pure (fetal and maternal DNA likely included). Samples were then processed to cDNA and run on a human TLR qPCR array (SABioscience, Valencia, CA). When three different monocyte populations were treated with the same cff-DNA sample, we found TLR3, TIRAP, IL-2, and Table 1 to have a fold change greater than two in all three populations compared to untreated controls (Figure 2A). TLR9, the known receptor for DNA binding, was downregulated in two out of the three samples suggesting endosomal degradation possibly due to incubating the cells with DNA too long. TLR3 is known to bind double stranded RNA, but was consistently impacted by our cff-DNA fraction, suggesting that either our fraction contains RNA or cff-DNA is similar enough to viral RNA that it can bind and activate TLR3 dependent inflammation. We repeated the same experiment using the same donor cells (44–year-old male) and tested three different cff-DNA samples isolated from different women near term. Although there were some differences based on the cff-DNA sample, we found similar genes to be changed twofold with treatment of cff-DNA (Figure 2B). This data can only illustrate the TLR effect of cff-DNA; further research is necessary to determine the role of fetal nucleic acids in pregnancy and the possibility that these short fragments could be used as a potential therapy for reactivating a proinflammatory response in immune tolerant cancer patients. Yet, the differential expression of genes involved in TLR signaling between cells treated with and without cff-DNA suggests that a mechanism may be worth pursuing.

## Conclusion

The role of the immune system in cancer progression and response to therapy has become increasingly appreciated in the past decade. Research into the development of chronic inflammation and the promotion of tumor growth has shed light on the need for immunologic intervention in order to cure cancer. Advancements in immunotherapies, including vaccines, cell therapy, and checkpoint inhibitors, have had exciting success; however, a majority of patients see little effect but still run the risk debilitating side effects. We have seen promise in using concepts from pregnancy for reactivating the proinflammatory immune response necessary for anti-tumor effects in melanoma patients. Specifically, a better understanding of the role of cff-DNA in the breakage of tolerance during late pregnancy may offer insight into predicting time of delivery in obstetrics and provide new ideas along with methods that may be applicable to cancer immunology. Further research into cell-free nucleic acids and TLR signaling could be the key to more effectively disrupting chronic inflammation in cancer patients and improving outcomes.

## Author Contributions

EE, WN, SH, and SM contributed to the ideas, concepts, and interpretations of this work and critically drafted/revised it for scientific integrity and accountability. Final draft was approved by all the authors. EE prepared the manuscript.

## Conflict of Interest Statement

The authors declare that this research was conducted without any commercial/financial relationships which could cause a conflict of interest.
